# Risk factors for mortality among drug-resistant tuberculosis patients registered for drug-resistant treatment in Amhara region, Ethiopia: a historical cohort study

**DOI:** 10.1186/s13690-020-00448-5

**Published:** 2020-07-31

**Authors:** Daniel Bekele Ketema, Muluneh Alene, Moges Agazhe Assemie, Leltework Yismaw, Mehari Woldemariam Merid

**Affiliations:** 1 Department of Public Health, College of Health Science, Debre Markos University, Debre Markos, Ethiopia; 2 Department of Epidemiology and Biostatistics, Institute of Public Health, College of Medicine and Health Sciences and Specialized Comprehensive Hospital, University of Gondar, Gondar, Ethiopia

**Keywords:** Risk factors, Drug-resistant tuberculosis, Mortality

## Abstract

**Background:**

The emergency of drug resistant tuberculosis is a major public health concern worldwide including Ethiopia. However, little is known about risk factors of mortality among drug resistant tuberculosis patients in the study site. Thus, this study was aimed to estimate the risks factors for mortality among drug resistant tuberculosis patients registered for drug-resistant treatment in Amhara region, Ethiopia.

**Methods:**

An institutional-based retrospective cohort study was carried out among laboratory-confirmed drug-resistant tuberculosis patients (*n* = 498) who were initiated treatment for drug-resistant tuberculosis between September 1, 2010, and December 31, 2017. The demographic and clinical characteristics of the patients were obtained from the register of patients treated for drug-resistant tuberculosis. The data were entered using EpiData version 4.2 and exported to Stata Version 14.1 for further analysis. Descriptive measures were used to characterize the study participants. Kaplan-Meier was used to estimate the survival time of the patients. Cox proportional hazard model was used to identify risk factors for mortality. Hazard Ratio (HR) with 95% confidence interval was used to report the strength of association between risk factors and mortality.

**Results:**

Death was observed among 14.2% (*n* = 70) of the 498 patients who met the inclusion criteria. The incidence rate of death was 8.20 (95% CI: 7.62, 20.50) per 1000 Person-months in the course of follow-up. The median age was 29.30 years (IQR:23–41). Age 45 years and above (adjusted hazard ratio (AHR) = 1.28: 95% CI: 1.10, 1.68), smoking cigarette (AHR = 1.39: 95% CI:1.27,3.18), tuberculosis related complication (AHR = 9.31:95% CI:5.11,16.97), anemia (AHR = 3.04:95% CI:1.14, 9.20), HIV/AIDS (AHR = 1.34:95% CI:1.25, 3.35), previous tuberculosis treatment history (AHR = 1.37:95% CI:1.16, 1.86), and diabetes mellitus (AHR = 1.85:95% CI:1.24,5.71) were identified risk factors for mortality.

**Conclusions:**

This study concluded that drug-resistant tuberculosis mortality remains high in the study site. Age 45 years and above, smoking cigarette, tuberculosis related clinical complication, being anemic at baseline, HIV/AID, previous tuberculosis treatment history, and diabetes mellitus were identified risk factors for mortality. Continual support of the integration of TB/HIV service with emphasis and working on identified risk factors may help in reducing drug-resistant tuberculosis mortality.

## Background

Tuberculosis (TB), a preventable and treatable disease, remains one of the important causes of death worldwide [1]. The World Health Organization (WHO), through the End TB Strategy [2], envisions the eradication of death, diseases, and suffering due to TB by 2035 [1]. However, Drug-Resistant TB (DR-TB) impede the realization of this vision [3] because of its lengthy, toxic, high cost of treatment, and poorer treatment outcome when compared to drug-susceptible TB. DR-TB is caused by TB bacteria that are resistant to at least one first-line anti-TB drug, while Multidrug-resistant TB (MDR-TB) is caused by TB bacteria that are resistant to at least isoniazid and rifampin, the 2 most potent TB drugs [1].

DR-TB remains major drug-resistant airborne infection and about one-third of deaths were attributed by DR-TB from antimicrobial resistance [4]. Overall, DR-TB causes about 10% of all TB deaths and it is a global threat [5, 6]. According to WHO 2017, global TB report, 600,000 DR-TB cases were estimated to be diagnosed globally and caused 240,000 deaths in 2016, and most deaths occurred in Asia and Africa region [7, 8]. Even though WHO has recommended shorter regimens (9–12-months) for specific groups of patients, majority DR-TB cases were treated for a minimum of 18–24 months with second-line TB drugs that have significant adverse effects [9].

A previous studies suggested that the prevalence of DR-TB was progressively increased in sub-Saharan Africa, where health resources, finances, and the skilled personnel required for diagnosis and management are limited [10–12]. The global data shows that only 54% of MDR-TB patients are successfully treated due to high mortality and lost follow up [7].

Ethiopia is one of the 30 high burden DR-TB countries identified by WHO with an estimated 5.8/1000 new MDR/RR-TB cases per year in 2016 [1] . A previous studies done at St. Peter’s specialised TB hospital in Addis Ababa, the capital city of Ethiopia, found that 15.30% known deaths with incidence rate of 3.6 per 10,000 person-days [13]. Other study conducted at University of Gondar specialized hospital demonstrated that 13.9% DR-TB patients were died and 5.9% lost follow-up in the entire follow up time [14]. Therefore, understanding risk factors for DR-TB mortality is vital to improving DR-TB treatment outcomes. Previous studies have shown that increasing age [15, 16], smoking cigarette [17, 18], comorbidities [19–21] such as HIV/AIDS and diabetes, clinical complication [14, 21, 22] therapeutic delay [3, 23, 24] were contributing factors for DR-TB mortality .

Despite of the approval of standard TB prevention and control program, Ethiopia remains one of the high TB and DR-TB burden countries where TB remains a substantial cause of morbidity and mortality [25, 26]. Knowledge on the risk of DR-TB mortality is critical for informing health policy solutions needed to improve the outcome of DR-TB care and contain the spread of disease. As mentioned above studies in other settings identified several factors for mortality of patients with DR-TB patients. However, only few studies have evaluated the risk factors for mortality in Ethiopia [13, 14, 18], and none in Amhara region by including all the major three DR-TB treatment initiating center (Gondar university specialized comprehensive hospital, Debre Markos Referral Hospital and Boru Meda Hospital). Therefore, the aim of this study was to estimate the risk factors for mortality among drug-resistant tuberculosis patients registered for drug-resistant treatment in Amhara region, Ethiopia.

## Methods

### Study design and settings

An institutional based retrospective cohort study was conducted among DR-TB patients who have commencing treatment from September 1, 2010 to December 31, 2017 in Amhara region. Among DR-TB treatment initiating centers found in the region, University of Gondar Comprehensive Specialized Hospital, Boru-Meda Hospital, and Debre-Markos Referral Hospital were the three treatment centers which provides a services for more than 80% of the DR-TB patients found in the region. University of Gondar Comprehensive Specialized Hospital, which is located in North Gondar Administrative Zone, the Amhara Regional State, started DR-TB treatment as a pilot program with the Global Health Commute (GHC) to treat patients as a national response to the emerging threat of drug-resistant TB in September 2010. The second setting is Boru-Meda Hospital which located in South Wollo zone, Amhara regional sate. The third study site is setting is Debre-Markos Referral Hospital, which was found in Debre Markos town, Amhara region.

### Sample size determination

All DR-TB patients who were enrolled at the University of Gondar Compressive Specialized Hospital, Debre Markos Referral Hospital and Boru-Meda Hospital from September 12,010 to December 31, 2017 were considered for this study. To check the efficiency, minimum adequacy of samples (427) was determined based on survival power formula as follow.

$$ {\displaystyle \begin{array}{c}E=\frac{Z\left(a/2\right)+ Z\beta}{p1\left(1-p1\right){(lnHR)}^2}\\ {}N=\frac{E}{p(E)}\end{array}} $$

Where: E = require number of events, p(E) = is average probability of event among exposed individuals from previous study, N = minimal sample size, p1 = Proportion of subjects under exposure variable, and HR = hazard ratio.

### Assumption

Two-sided Z value at 95% confidence interval = 1.96, Power = 80%, β = 0.2, Zβ = 0.842.

Accordingly:

**Table:** 

Variables	HR	P1	Event	Pro (event)	Sample size
HIV co infection [14]	2.6	0.217	53	0.23	230
Anemia [27]	2.2	0.37	56	0.2	281
Drug susceptibility [18]	2.33	0.21	66	0.178	371

Accordingly, the maximum sample size based on the above formula after considering 15% incompleteness was 427. However, the actual data collected in a study area were 498 which maximize the true estimate of parameters.

### Study population

We included all patients, who had laboratory confirmed DR-TB, and registered on DR-TB register book and started treatment between September 12,010 and December 31,2017.

### Exclusion criteria

Patients who had not been assigned a treatment outcome at the end of the follow-up period, clinically diagnosed cases (non-laboratory confirmed cases) and transferred in cases from other health facilities were excluded from the analysis.

### Variables of the study

Death, as defined by the national TB program, was the main outcome of this study. The Ethiopia TB programme, in accordance WHO classification, “death” as any patient who dies during the course of DR-TB treatment [7].

Independent variables considered in the analysis included (age, sex, anti-TB drug history, marital status, level of education, religion) and clinical (HIV status, diabetes mellitus, hypertension, asthma, types of resistant, initial smear and culture result) and behavioural (cigarette smoking).

### Definition of variables

DR-TB treatment outcomes were assigned as per the definitions in the Ethiopian national TB guidelines, which have been adopted wholly from the WHO definitions and reporting framework for TB guidelines as cured, treatment completed, treatment failed, died, lost to follow-up and not evaluated [28].

#### Cured

patients completed treatment without evidence of treatment failure and had three or more consecutive negative culture taken 30 days apart.

#### Treatment complete

complete treatment without evidence of treatment failure but no recorded culture result.

#### LTFU

Patients who interrupt treatment 2 or more consecutive months.

#### Death

Referred to death for any reason during the course of treatment.

#### Time to death

Is the time gap, in months, between the beginning of 190 DR-TB treatment and the date of death.

#### Censored

If they had the TB treatment outcome of cured, completed, and transferred out or lost to follow-up or were still on treatment at the end of the study.

#### Anemia

Based on the WHO definition, patients were considered as anemic if their haemoglobin level < 12 g/dl for female and children and less than 13 g/dl for men.

### Source of data and procedure

This study used secondary data that were collected using a structured data extraction checklist. Data were extracted from patients’ DR-TB registration books, treatment follow-up sheet, green card and medical records. These data sources have contained a sociodemographic characteristic (age, sex, residence, marital status, educational status, occupation, religion), clinical variables (HIV status and other comorbidities, site of TB disease, number of previous TB treatments, initial DR-TB regimen, initial regimen change, initial sputum and culture result). Data were collected by workers who were working in the DR-TB treatment centre.

### Data quality management

Training on the objective of the study and how to retrieve records as per data extraction sheet was given to data collectors and supervisors for 2 days before data collection started. The data extraction sheets were pre-tested for consistency of understanding of tools and completeness of data for charts. Necessary adjustment for the final data collection sheet was made.

### Data processing and analysis

The data were checked for inconsistencies, coding error, completeness, accuracy, clarity, and missing values before they were entered. The data were entered using EpiData version 4.2 and exported to Stata Version 14.1 for further analysis. Descriptive measures were used to characterize the study participants. The time for death was estimated using the Kaplan-Meier method. The log-rank test was used to compare hazard curves between baseline categorical variables. Incidence of death with respect to person time at risk was calculated. Variables which are significant at *P* < 0.20 in the bivariable analysis were included in the final Cox- regression analysis. Proportionality assumption was tested by global test based on scheonfeld residuals. Hazard ratio and 95% confidence interval were used to report the strength of association between mortality rate and it's risk factors.

## Results

### Baseline demographic and clinical characteristics

A total of 565 patients were registered for DR-TB treatment between September 1, 2010 and December 31, 2017. We analysed data from 498 laboratory confirmed DR-TB patients, after excluding patients who were clinically diagnosed (*n* = 15), transferred in from other health facility (*n* = 35) and patients who had not been assigned a treatment outcome at the end of the follow-up period (*n* = 17). Among a total of 498 study participants, almost half 261 (52.4%) were male (Table 1).

**Table 1:** Socio-demographic characteristics of drug-resistant tuberculosis patients in Amhara region, from September 1, 2010 to December 31, 2017

Variables	Frequency	Percent (%)
**Sex**		
Male	261	52.4
Female	237	48.6
**Marital status(*n*** **= 494)**		
Never married	248	50.2
Married	183	37.1
Divorced	47	9.5
Widowed	16	3.2
**Level of education**		
No education	222	44.6
Primary	137	27.5
Secondary and above	139	27.9
**Residence**		
Urban	238	47.8
Rural	260	52.2

The median age of DR-TB patients was 29.30 years [IQR:23–41 years]. The length of hospital stay was higher for patients who were died compared to patients who did not with median of 63 days and 54 days respectively. The median body mass index (BMI) of DR-TB patients at baseline was 17.51 Kg/m^2^ [IQR: 15.82–21.00] (Table 2).

**Table 2:** Descriptive Statistics of continuous covariates of drug-resistant tuberculosis patients in Amhara region, from September 1, 2010 to December 31, 2017

Variables	Median [IQR]
Age (Year)	29.50 [23,41]
BMI (Kg/m^2^)	17.51 [15.82,21.00]
Hospital stay in day	63 [30., 90]
Number of previous treatment	2 [1, 3]
Follow up time in month	20 [9, 21]

*BMI* Body Mass Index, IQR Inter Quartile Range

**Fig. 1:**
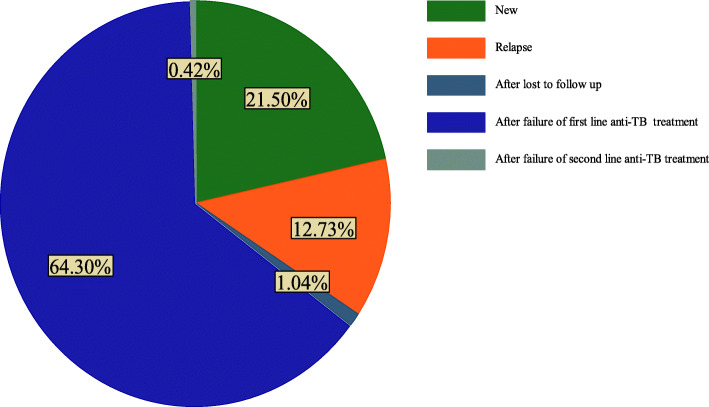
Registration group of drug-resistant tuberculosis patients in Amhara region, from September 1, 2010 to -December 31, 2017

About 421 (84.5%) patients had pulmonary TB, of which 79.3 and 96.4% patients had positive sputum smear and positive culture result at baseline respectively. With regard to drug-resistant patterns, half (50.4%) of patients were mono resistant, 41.2% were multidrug-resistant, 8.2% poly-resistant, and 0.2% extensive drug-resistant (Table 3). Among 498 DR-TB patients, nearly two third 320 (64%) of patients were enrolled after failure of first line anti-TB treatment followed by newly diagnosed cases 107 (21.5%) (Fig. 1).

**Table 3:** Baseline clinical characteristics of drug-resistant tuberculosis patients in Amhara region, from September 1, 2010 to December 31, 2017

Variables	Frequency	Percent (%)
**Baseline sputum smear**		
Positive	395	79.3
Negative	103	20.7
**Body mass index**		
< 18.5	383	76.9
≥ 18.5	115	23..1
**Diabetes**		
Yes	16	3.2
No	482	96.8
**HIV/AIDS**		
Positive	123	24.7
Negative	375	75.3
**Anaemia**		
Anemic	387	77.7
Non anemic	111	22.3
**Previous tuberculosis treatment history**		
Yes	399	80.1
No	99	19.9
**Tuberculosis type**		
Pulmonary	442	88.7
Disseminate	56	11.3
**Smoking cigarette**		
Yes	72	14.5
No	426	85.5

### Treatment outcomes and survival status

**Fig. 2:**
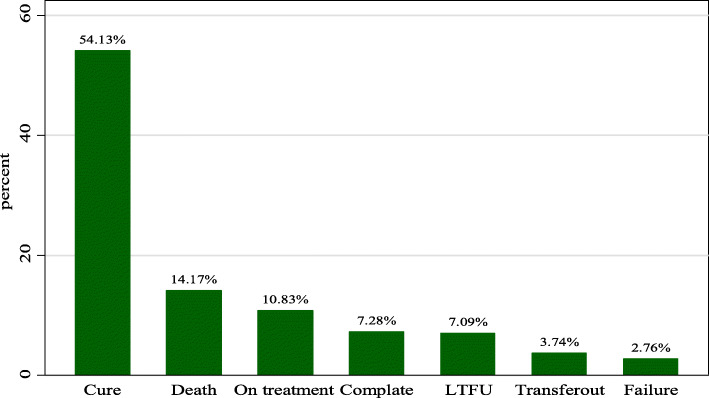
Treatment outcome of drug-resistant tuberculous patients in Amhara region, from September 1, 2010 to December 31, 2017

**Fig. 3:**
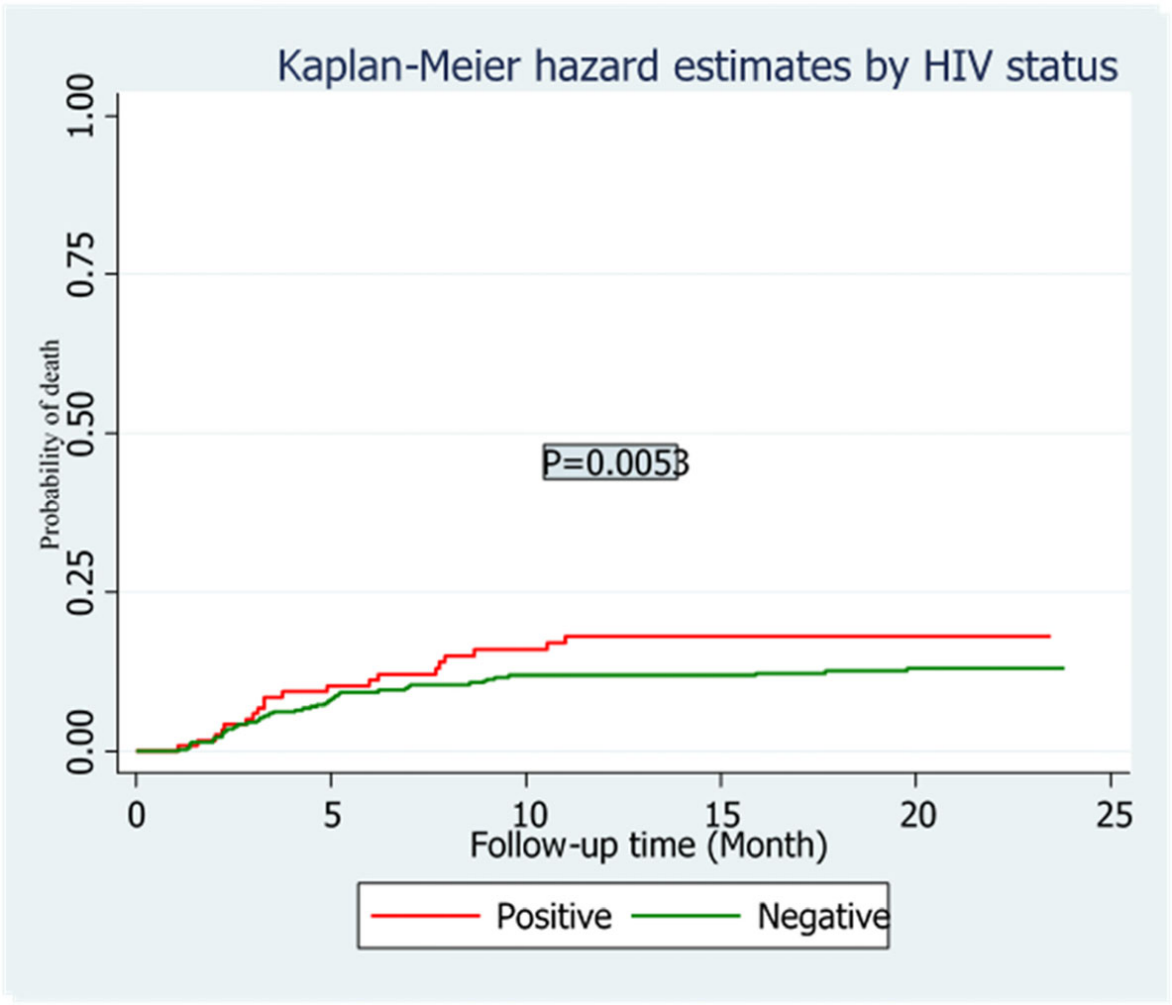
Kaplan-Meier hazard curve and Log Rank test by HIV status for drug-resistant tuberculosis patients in Amhara region from September 1, 2010 to December 31, 2017

**Fig. 4:**
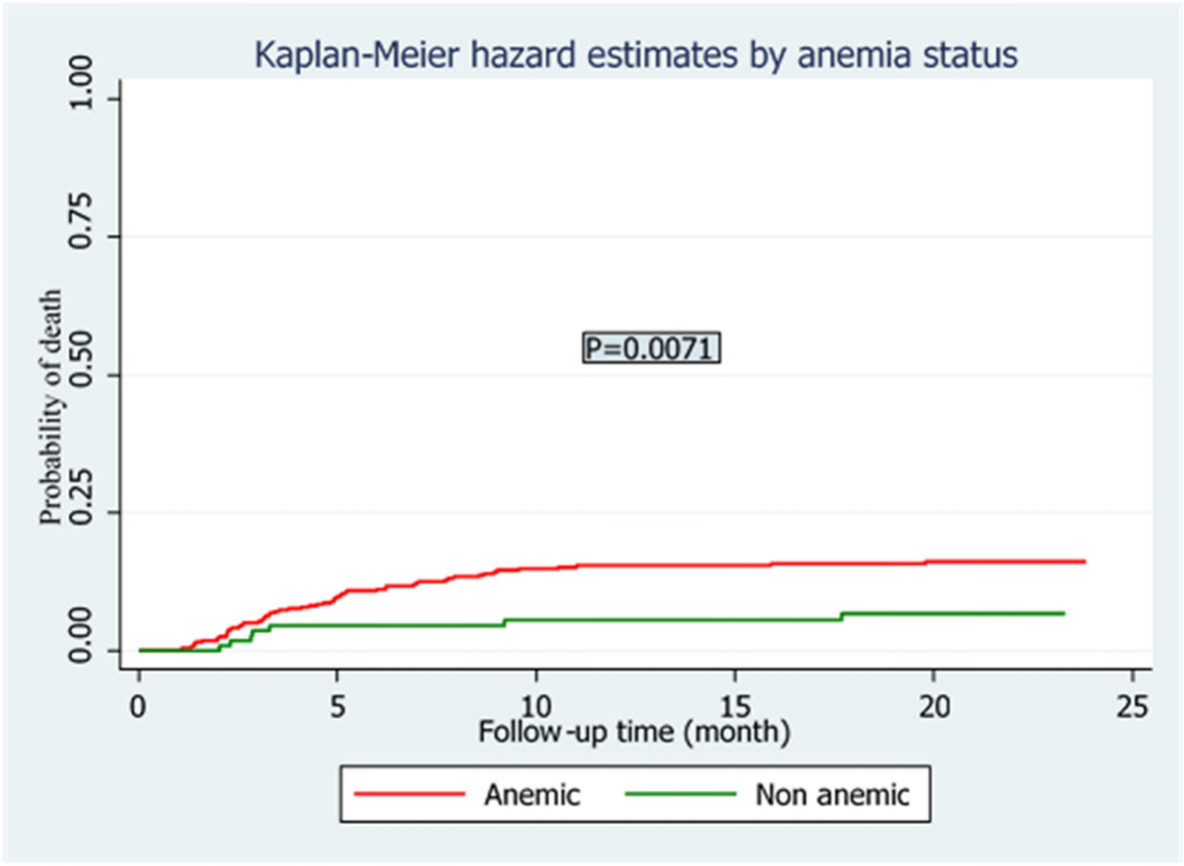
Kaplan-Meier hazard curve and Log Rank test by the level of haemoglobin (anemic vs no anemic) of drug-resistant tuberculosis patients in Amhara region from September 1, 2010 to December 31, 2017

The overall treatment success rate of 498 study participants was 61.4% (cure 54.1% and treatment completion 7.3%). In addition, in the follow up time,70 (14.1%) died, 14 (2.8%) had treatment failure,36 (7.1%) were lost to follow-up in the course of treatment (Fig. 2). The overall incidence density of death in the cohort was 8.20 (95% CI: 7.6, 20.5) per 1000 Person-months. Higher mortality rate was observed among individuals with HIV/AIDS with incidence rate of 7.4 per 1000 person-months compared to 1.1 per 1000 person-months among HIV negative patients. The log rank test also showed that the hazard rate of mortality was significantly higher among HIV positive patients as compared to HIV negative patients (Fig. 3). Based on the patient’s level of haemoglobin (categorized as Anemic and non anemic), the hazard of mortality was worsened for anemic patients as compared to non anemic patients in the course of follow-up time (Fig. 4).

### Predictors of DR-TB mortality

In the multivariate cox proportional hazard model, Age 45 years and above, smoking cigarette, tuberculosis related clinical complication, being anemic at baseline, HIV/AID, previous tuberculosis treatment history, and diabetes mellitus were significant risk factors for mortality (Table 4).

**Table 4:** Multivariable Cox regression analysis for risk factors for mortality among drug-resistant tuberculosis patients in Amhara region, from September 1, 2010 to December 31, 2017

Variables	CHR [95% CI]	AHR [95% CI]
**Age in years**		
< =24	1.00	1.00
25–44	0.42 [0.21 0.88]	0.45 [0.14 1.84]
45 and above	1.18 [1.12 1.43]	1.28 [1.10 1.68]
**Tuberculosis related complication**		
No	1.00	1.00
Yes	11.94 [7.14 19.97]	9.31 [5.11 16.97]
**Baseline sputum smear**		
Positive	1.00	1.00
Negative	1.56 [0.75 3.27]	1.23 [0.65 2.33]
**Level of education**		
No education	2.69 [1.34 5.39]	1.29 [0.62 2.69]
Primary	1.67 [0.75 3.68]	1.66 [0.74 3.70]
Secondary and above	1.00	1.00
**Anaemia**		
Non anaemic	1.00	1.00
Anaemic	[1.35 8.41]	3.04 [1.14 9.20]
**HIV/AIDS**		
Negative	1.00	1.00
Positive	1.42 [1.21 4.84]	1.34 [1.25 3.35]
**Diabetes mellitus**		
No	1.00	1.00
Yes	2.52 [2.14 7.31.]	1.85 [1.24 5.71]
**Previous tuberculosis treatment history**		
No previous treatment history	1.00	1.00
Had previous treatment history	1.57 [1.22 1.79]	1.37 [1.16 1.86]
**Smoking cigarette**		
No	1.00	1.00
Yes	1.89 [1.04 3.36]	1.39 [1.27 3.18]

## Discussions

The objective of this study was to estimate the risk factors for mortality among DR-TB patients in Amhara region: Ethiopia. This study found that incidence of mortality was 8.2 per 1000 person-months observation. This finding was comparable with previous studies conducted in Ethiopia [14, 18], and other countries in sub-Saharan Africa [23]. In contrary, our finding was higher compared to the research conducted in St. Peter specialized TB hospital in Addis Ababa which is 3.6 per 10,000 person-days [13]. This difference may be the previous study only include DR-TB cases enrolled in one DR-TB treatment initiating center and only 188 DR-TB cases were included in the analysis.

Our study determined that being aged 45 years and above was a significant risk factor for mortality. This finding was consistent with findings reported in Eastern Cape Province, South Africa [29]. This evidence was supported by previously existing literature [30] which indicated that increased age is associated with increasing co-morbidities as well as weakening of body functions. As age increased the prevalence of comorbidities such as diabetes were also increased and parallel the risk of DR-TB mortality increased.

The study found that smoking cigarette was associated with mortality. This finding was in agreement with other studies elsewhere [17, 18]. Probably cigarette smoking speculated to lower cytokine-producing macrophages with diminished influx of interferon gamma producing effector T-cells in the lungs, which leads to increase the incidence of active and latent pulmonary TB as well poor clinical prognosis.

The study determined that HIV co-infection was a risk factor for mortality. This finding is consistent with previous studies conducted in Ethiopia [13, 18, 23]. This is due to HIV co-infection reduced the integrity and function of CD4+ cells which reduced the level of immunity and increased risk of mortality [31, 32] In addition, TB hand increases HIV replication and viral diversification rates, by increasing pro inflammatory cytokine production, which increase HIV viral replication and diversity, which in turn facilitating immune escape [33, 34]. This synergistic effect may be the principal reason for the high mortality among HIV confected DR-TB patients.

In this study patients who had a tuberculosis-related clinical complications have high risk of mortality. The presence of clinical complication were the sign of poor progression of diseases and subsequent increase in the risks of mortality. This finding was in consistent with studies conducted in Ethiopia [13, 14, 18].

This study also demonstrate that anemia considerably increase the risk of mortality when compared to non-anemic patients. This might be related with, anemic patients may liable to adverse drug effect during anti-tuberculosis treatment. This finding was comparable with other study which found that anaemia is associated with drug-resistant tuberculosis mortality [35]. In addition, anemic patients might have increased risks of infection and have compromised immunity which contribute for advancement of disease progression and risk of death.

Drug-resistant tuberculosis patients with diabetes mellitus co-morbidity have increased risk for mortality. This association has been demonstrated in the previous studies [20, 21, 36]. A possible justification might be, patients with diabetes mellitus have impaired immunity compared to healthy individuals, and sequel of diabetes may potentiate the adverse effects of anti-tuberculosis drugs.

### Limitation of the study

There was a limitation for this study. Since the study was based on secondary data; potential important variables such as radiograph findings and behavioural factors were not assessed to define the best predictors of mortality. Other limitation of this study was non-tuberculosis related death such as accidents or other chronic diseases my cause death in the course of treatment, but specific cause of death was not available. This may lead to over estimation of the incidence of death rate in our study. Therefore, is recommended to computing risk analysis for each specific cause of death in the course of treatment.

## Conclusions

This study concluded that drug-resistant tuberculosis mortality remains high in the study site. Age 45 years and above, smoking cigarette, tuberculosis related clinical complication, being anemic at baseline, HIV/AID, previous tuberculosis treatment history, and diabetes mellitus were identified risk factors for mortality. Continual support of the integration of TB/HIV service with emphasis and working on identified risk factors may help in reducing drug-resistant tuberculosis mortality.

## Data Availability

The datasets used and/or analysed during the current study are available from the corresponding author on reasonable request.

## Abbreviations

DR-TB: Drug Resistant Tuberculosis
MDR-TB: Multidrug Resistant Tuberculosis,
HIV: Human immune Deficiency Virus
WHO: World Health Organization
DOTS: Direct observed treatment, short course
DM: Diabetes Mellitus
AIC: Akaike information criteria
